# Measuring adherence, acceptability and likability of an artificial-intelligence-based, gamified phone application to improve the quality of dietary choices of adolescents in Ghana and Vietnam: Protocol of a randomized controlled pilot test

**DOI:** 10.3389/fdgth.2022.961604

**Published:** 2022-12-06

**Authors:** Bianca C. Braga, Alejandra Arrieta, Boateng Bannerman, Frank Doyle, Gloria Folson, Rohit Gangupantulu, Nga Thu Hoang, Phuong Nam Huynh, Bastien Koch, Peter McCloskey, Lan Mai Tran, Trang Huyen T. Tran, Duong Thuy T. Truong, Phuong H. Nguyen, David Hughes, Aulo Gelli

**Affiliations:** ^1^ Friedman School of Nutrition Science and Policy, Tufts University, Boston, MA, United States; ^2^ Department of Health Metrics Sciences, University of Washington, Seattle, WA, United States; ^3^ Poverty, Health, and Nutrition Division, International Food Policy Research Institute, Washington, DC, United States; ^4^ Department of Nutrition, Noguchi Memorial Institute for Medical Research, College of Health Sciences, University of Ghana, Legon, Ghana; ^5^ Department of Entomology, Pennsylvania State University, University Park, PA, United States; ^6^ National Institute of Nutrition, Ha Noi, Vietnam; ^7^ Hubert Department of Global Health, Rolling School of Public Health, Emory University, Atlanta, GA, United States; ^8^ Thai Nguyen University of Medicine and Pharmacy, Thai Nguyen, Vietnam; ^9^ Department of Biology, Pennsylvania State University, University Park, PA, United States

**Keywords:** dietary assessment method, behavior change app, artificial intelligence - AI, food choice, digital food environment

## Abstract

Unhealthy diets are a critical global concern while dietary measure methods are time consuming and expensive. There is limited evidence that phone-based interventions can improve nutrition data collection and dietary quality, especially for adolescents in developing countries. We developed an artificial-intelligence-based phone application called *Food Recognition Assistance and Nudging Insights (FRANI)* to address these problems. *FRANI* can recognize foods in images, track food consumption, display statistics and use gamified nudges to give positive feedback on healthy food choice. This study protocol describes the design of new pilot studies aimed at measuring the feasibility (acceptability, adherence, and usability) of *FRANI* and its effects on the quality of food choice of adolescents in Ghana and Vietnam. In each country, 36 adolescents (12–18 years) will be randomly allocated into two groups: The intervention group with the full version of *FRANI* and the control group with the functionality limited to image recognition and dietary assessment. Participants in both groups will have their food choices tracked for four weeks. The control groups will then switch to the full version of *FRANI* and both groups will be tracked for a further 2 weeks to assess acceptability, adherence, and usability. Analysis of outcomes will be by intent to treat and differences in outcomes between intervention and control group will use Poisson and odds ratio regression models, accounting for repeated measures at individual levels. If deemed feasible, acceptable and usable, *FRANI* will address gaps in the literature and advance the nutrition field by potentially improving the quality of food choices of adolescent girls in developing countries. This pilot study will also provide insights on the design of a large randomized controlled trial. The functioning and dissemination of *FRANI* can be an important step towards highly scalable nutrition data collection and healthier food choices for a population at risk of malnutrition.

The study protocol and the methods and materials were approved by the Institutional Review Board (IRB) of the IFPRI on April 29th, 2020 (registration number #00007490), the Thai Nguyen National Hospital on April 14th, 2020 (protocol code 274/ĐĐĐ-BVTWTN) and the University of Ghana on August 10th, 2020 (Federalwide Assurance FWA 00001824; NMIMR-IRB CPN 078–19/20). The study protocol was registered in the International Standard Randomized Controlled Trial Number (ISRCTN 10681553; https://doi.org/10.1186/ISRCTN10681553) on November 12, 2021.

## Introduction

There are 1.8 billion adolescents in low- and middle-income countries (LMIC) (1). Adolescence is characterized by rapid physical growth, sexual maturation, and increased nutrient requirements (2). Meeting nutrient requirements is essential to optimize biological development, growth, long-term health, and longevity (2–4). Those who complete the physical, mental, social and emotional development of adolescence have better chances to reproduce successfully and upbring their children optimally (2). Investments to end malnutrition during this life stage are a great opportunity to interrupt intergenerational cycles of malnutrition and improve child and maternal health (5, 6).

Every year, around 16 million adolescent girls become mothers in LMIC (7). The absolute number of adolescent pregnancies is increasing, especially in settings of high prevalence of malnutrition, food insecurity and poor dietary quality such as Africa, South Asia and Southeast Asia (2). Both under and overnutrition before and during pregnancy predict altered growth and health of the offspring (8). Pregnant adolescents typically compete for nutrients with the fetus, increasing risk of low birthweight and short birth length (9, 10). The risk of preterm delivery, and poor childhood growth and nutritional status is also higher for neonates of adolescent mothers (11, 12).

Solutions to maternal and child malnutrition in LMIC have to take into account the social and cultural factors that drive adolescent decision making (5, 13, 14). Adolescent neuro-development make them more aware of external influences, preferences and habits of peers and family (15, 16). This increases the desire to have social recognition, status, and autonomy (17–19). Responsibility over food acquisition, preparation, and consumption start in adolescence (6). The sensitivity to external influence coupled with greater power for food choice (20) create a unique opportunity to foster long-term healthy eating (6).

Data collection for adolescent nutrition is fundamental to design effective policies and programs and to assess their progress (5, 6, 13). The Nudging for Good project aims at developing, validating and examining the feasibility of the Food Recognition Assistance and Nudging Insights (FRANI), a phone application that uses artificial intelligence technology to recognize foods in pictures, assess diets and nudge adolescents from Ghana and Vietnam to make healthier food choices (21). First, we made food inventories, prepared and cooked these foods, weighted, annotated and took pictures of them. We linked the pictures to food composition tables, and built models to recognize the images and estimate portion sizes. Finally, we developed the interface according to the preferences showed in focus groups discussions by adolescent girls from Ghana and Vietnam. We validated the FRANI dietary assessment through food image recognition against the gold standards of weigh food records and 24 h recalls. Now, we aim at conducting a randomized controlled pilot study to measure the feasibility of using FRANI and its impact on the quality of food choice.

### The intervention

The FRANI mobile app is designed to improve diets by increasing the consumption of healthy foods and beverages, whilst crowding out the consumption of energy-dense foods. FRANI users can set healthy eating goals – or choose daily dietary goals from a list of simple options based on food groups, such as “have a dark green vegetable” or “eat a whole grain”. Conceptually, this increases motivation and interest in learning about healthy diets, as well as increases awareness of what are healthy and unhealthy foods. Alongside individual-based goals, users can opt-in to team-based goals that may lead to stronger peer support on healthy eating practices and provide social praise for healthier food choices. However, in order to avoid peer-pressure, users can only visualize team progress as a whole and cannot track the individual performance of peers.

FRANI provides feedback based on the information uploaded by users, or on the pictures of foods and beverages they consume. The AI technology is trained on a database of calibrated images with foods and beverages to enable estimation of both the foods and portion sizes in the uploaded pictures. FRANI then coverts the food quantities into nutrients by using food composition tables. The practicality of this technology has the potential to increase the frequency of data points collected on food consumption, addressing the constraint faced by common diet estimation methods with regards to estimating usual intake (22).

## Methods and analysis

### Materials

**Figure 1:**
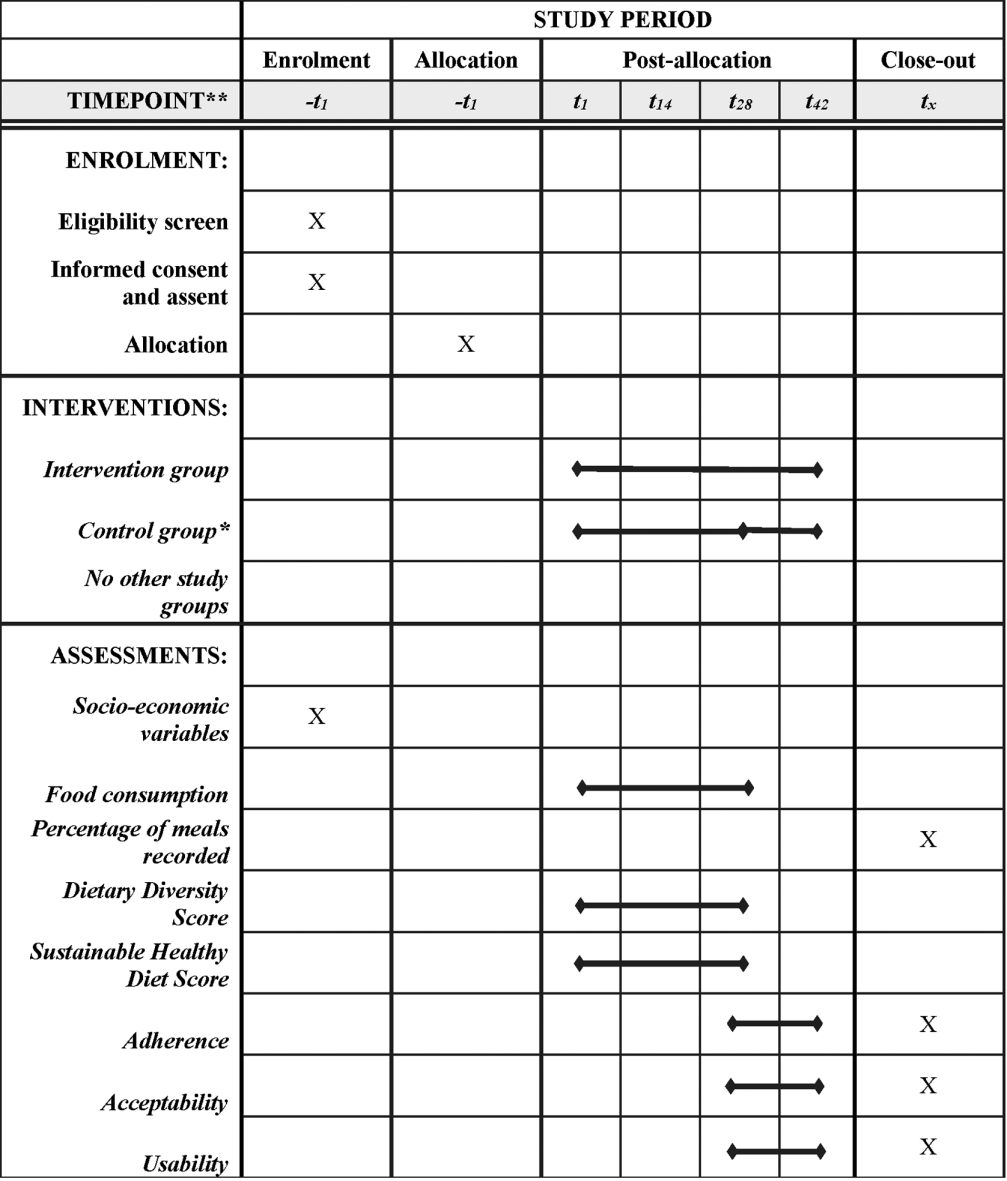
Adapted SPIRIT diagram (29). Schedule of enrolment, interventions, and assessments of the two-armed randomized controlled pilot studies in Ghana and Vietnam. *The participants in the control group will use FRANI with limited functionality to track their food choices for 28 days (4 weeks). After that, the control group will switch to also use FRANI with full functionality for the last two weeks of the post-allocation period. The bullet in t28 indicates that the control group switched for the full version app.

**Figure 2:**
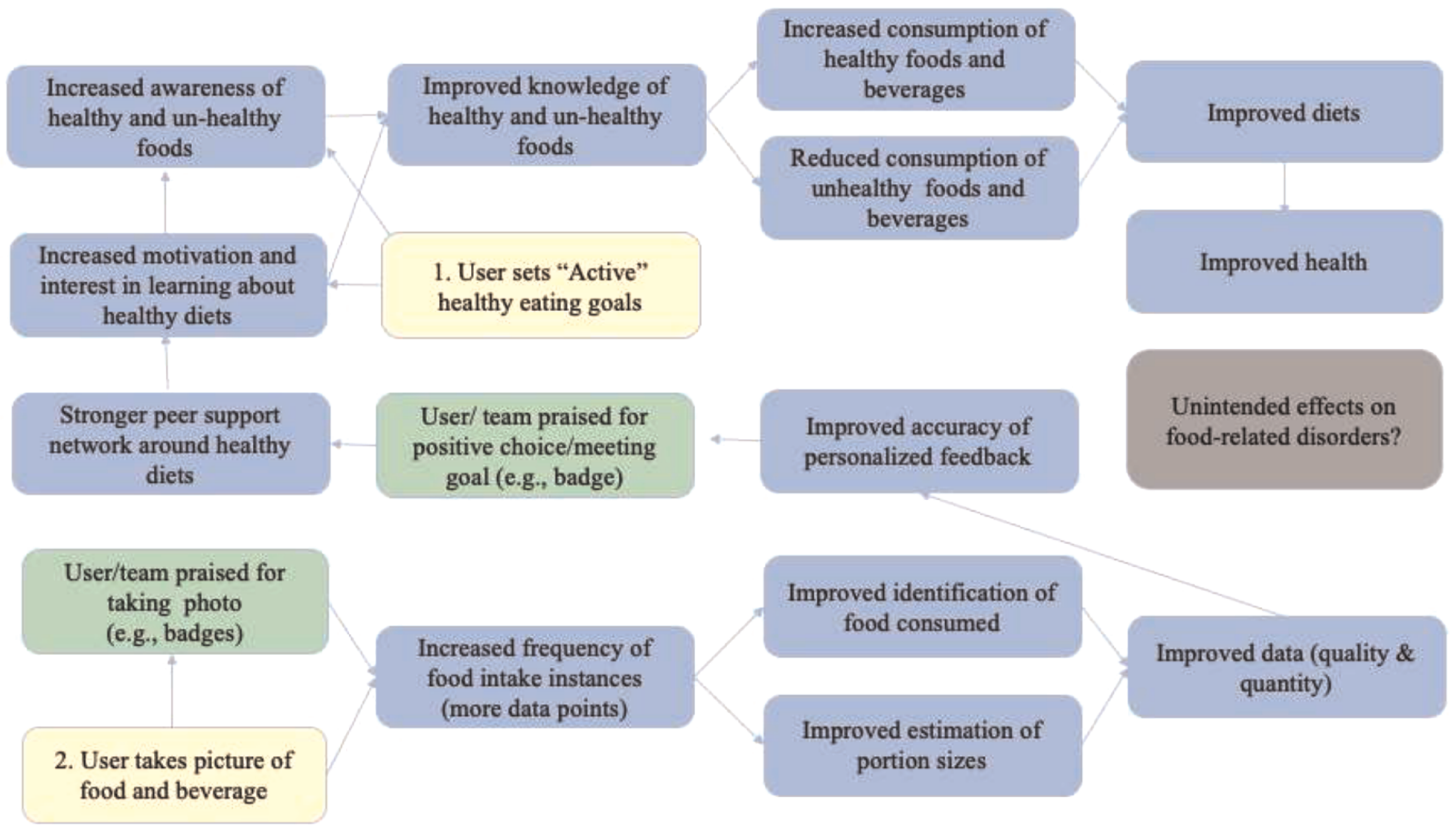
The stylized program impact pathways for the FRANI. (Source: Adapted from (21)) Notes: Yellow represents the two steps users have to follow to have their dietary consumption tracked and to be nudged to make healthier food choices. Green boxes show nudges to influence the use of the FRANI and healthy food choices. Blue boxes represent what is expected to improve with the FRANI use. Gray is for a possible unintended consequence of the FRANI use that should be fixed in future versions of the app.

The pilot studies are based on two-armed randomized controlled designs to be implemented in Ghana and Vietnam. In each country, 36 adolescents (12–18 years) will be randomly allocated into two groups: The intervention group with the full version of FRANI, including diet records and nudges to encourage healthier food choices. The control group will receive mobile phones with FRANI functionality limited to the dietary assessment. Participants in both groups will have their food choices tracked for a four week period. The control group will then switch to also use FRANI with full functionality and both groups will be tracked for a further 2 weeks to assess acceptability, adherence, and usability. The schedule of the intervention, enrollment and assessment is described in Figure 1. The stylized program impact pathway for the intervention is described in Figure 2 (21).

The main differences in the application versions for the intervention and control groups are described in Table 1. Briefly, both the intervention and control versions of FRANI recognize food images, assess the quality of the user's diet, and receive notifications with reminders to take pictures. However, only the treatment group can set food consumption goals, see the home screen, scores and statistics about dietary quality, receive badges and receive a daily report summarizing everything they ate in that day.

**Table 1:** Features of FRANI by intervention group in the first four weeks of the pilot study.

Feature	Intervention	Control
Meal entry (picture taking)	X	X
Recognize food image in pictures	X	X
Assess quality of user's diet	X	X
Notifications	X	X
Setting goals	X	NA
Home screen	X	NA
Scores and statistics	X	NA
Medals and badges	X	NA
Daily report	X	NA
Activity feed	X	Modified
Daily report	X	NA

NA, not available. At the end of the fourth week, the control group switches to the intervention version and all participants use the same version of FRANI for two weeks to measure adherence, acceptability and usability.

The options for setting food consumption goals were based on the EAT-Lancet Diet (23) and the Dietary Guidelines of Vietnam (24). These FRANI goals can be individual- or team-oriented, and involve five main food groups: (1) grains and starchy staples; (2) legumes, nuts and seeds; (3) fruits and vegetables; (4) dairies; (5) animal-source foods (excluding dairy). In addition to setting food consumption goals, participants from the intervention group be nudged with game elements. These nudges include: (1) tracking progress towards healthy-eating goals; (2) giving personalized feedback on food consumption; (3) reinforcing healthy food consumption; (4) tracking progress in the quality of food consumption with statistics; (5) participating in goals shared with other users (team-based goals); (6) sharing positive feedback with peers. Participants in the intervention group will be able to choose from team-based goals on a weekly basis. They will receive information about their team's overall performance but in order to avoid excessive peer pressure they will not be able to see each individual team member's performance.

The study protocol and the methods and materials were approved by the Institutional Review Board (IRB) of the IFPRI on April 29th, 2020 (registration number #00007490), the Thai Nguyen National Hospital on April 14th, 2020 (protocol code 274/ĐĐĐ-BVTWTN) and the University of Ghana on August 10th, 2020 (Federalwide Assurance FWA 00001824; NMIMR-IRB CPN 078–19/20). The study protocol was registered in the International Standard Randomized Controlled Trial Number (ISRCTN 10681553; https://doi.org/10.1186/ISRCTN10681553) on November 12, 2021. Participants will be instructed to report any problem occurring during the study to the country-lead researchers, which will be in direct contact with them. Although risk from using FRANI is minimal, any adverse effect will be discussed by the team, and reported to the IRB of the IFPRI and to the ethics boards of correspondent countries. All procedures were and will be in accordance with the Declaration of Helsinki.

### Study sites

The FRANI pilot test is targeted to adolescent girls from 12 to 18 years old living Accra, Ghana and Thai Nguyen, Vietnam. These sites were selected based on relevance of the dietary quality concern and proximity to research partners.

### Inclusion and exclusion criteria, and recruitment

A total of 72 adolescents girls aged between 12 and 18 years will be randomly selected from two schools, one in each city, which staff has a good working relationship with our team. Those who assented for participation and which parents consented will receive a smartphone pre-configured with the FRANI app. Participants will be free to discontinue study participation at any time. Candidates will be excluded from the study if they do not provide assent or their parents do not provide consent to participate.

### Randomization and allocation

A computer-generated random number will randomize participants in the intervention and control groups at a 1:1 ratio for each country separately. The allocation sequence will be generated and the enrollment and assignment will be done by the senior researchers of the Vietnamese and Ghanaian teams. Although a similar description of FRANI will be presented for intervention and control groups in the process of informed assent and consent, it will not be possible to blind who is receiving feedback from FRANI and who is not. The data will be collected by the application without assessors.

### Outcomes

The feasibility of FRANI will be assessed by measuring outcomes related to adherence, acceptability, and usability as summarized in Table 2 (25, 26). According to the World Health Organization (WHO), adherence is “the extent to which a person's behavior – taking medication, following a diet, or executing lifestyle changes, corresponds with agreed recommendations from a health care provider” (27). Although there is no consensus in the literature on the best measure for adherence to eHealth technology interventions (25, 26), maximum benefit from the intervention as defined by creators should be considered (28). We define the sample adherent to FRANI if 70% or more of participants-days upload complete dietary recalls in the last two weeks of the pilot study. Acceptability summarizes likeability and satisfaction with FRANI, while usability summarizes what affects the use of the FRANI. Acceptability and usability are measured according to answers from structured questionnaires described in Table 3. If participants grade the acceptability or usability of FRANI as 30 or less points in their respective structured questionnaires, the app will be considered accepted or usable.

**Table 2:** Domains and outcome measures for the FRANI pilot test.

Outcomes	Definition
Primary outcomes (feasibility)	
Adherence	The number of days participants upload completed dietary recalls on the FRANI divided by the total number of dietary recalls (complete + incomplete) of the last two weeks of the pilot test is equal to or higher than 70%
Acceptability	Summarizes likeability and satisfaction with FRANI according to answers from a structured questionnaire. If participants grade the acceptability of FRANI as 30 or less points on a scale from 10 to 50 points, the app will be considered accepted.
Usability	Summarizes possibility to use and intent to keep using FRANI according to answers from a structured questionnaire. If participants grade the usability of FRANI as 30 or less on a scale from 10 to 50 points, the app will be considered usable.
Secondary outcomes	
Percentage of meals recorded	The number of meals recorded on the FRANI divided by the number of total meals participants had during the last two weeks of the pilot test period.
Dietary Diversity Score^a^	Ranges from 0 to 10 points using MDDW dietary diversity food groups (daily score) measured in the first four weeks of the pilot study period.
Sustainable Healthy Diet Score^b^	Ranges from 0 to 14 points using EAT-Lancet food groups (daily score) measured in the first four weeks of the pilot study period.

^a^ The dietary diversity score was calculated according to the Minimum Dietary Diversity for Women, A Guide for Measure (26).

^b^ The Sustainable Healthy Diet Score was calculated according to the study EAT- Lancet Score and Major Health Outcomes: The EPIC-Oxford Study (25).

**Table 3:** Questionnaire about acceptability and usability of the FRANI application.

**Acceptability**
I like this app
I want to keep using this app
This app helps me to eat healthy
I like receiving badges
I like the daily diet reports
I like the daily statistics page
I like the weekly statistics page
I like following other users
I like to be part of a team
I like the general navigation of the screens
**Usability**
This app is simple to understand
I felt comfortable using this app
I felt praised when I ate healthy foods
I felt supported by my app friends
I can take pictures of my meals at home
I can take pictures of my meals in school
I can take pictures of my meals when I am eating with my family
I can separate what I eat from what others are eating to take pictures
I trust this app's feedback about my diet
I trust this app's information about healthy eating

Responses are in Likert scale from 1 = strongly agree to 5 = strongly disagree.

Based on the analysis of the program theory, the effects of the FRANI and gamified nudges on the dietary quality of adolescent girls will be assessed on a set of secondary outcomes including the percentage of meals recorded and the quality of food choices. The quality of food choices will be measured according to the Dietary Diversity Score (DDS) and the Sustainable Healthy Diet Score (SHDS). Adherence, percentage of meals recorded, the DDS and the SHDS will be measured using data captured by FRANI. Although the secondary outcome measures will be based on intervention and control group comparisons, the primary measures will not.

The data for outcomes will be recorded on FRANI and uploaded to a data collection platform at real time during the intervention period. Data collection will happen automatically once the participants took pictures of the foods they ate and confirmed their recognition. Since the image recognition technology from FRANI has been shown to be promising in the validation study (not published yet), the automatization of the data entry process may minimize mistakes if compared to other forms of food consumption data collection such as 24 h recalls and food frequency questionnaires. Baseline variables such as parents occupation, household assets and school performance will be collected with questionnaires. The data will be stored in protected clouds at the Pennsylvania State University.

### Sample size

The sample size was determined by the number of smartphones and data packages that could be supported by budget and is consistent with other pilot studies in the literature. The effect size of the treatment on the DDS and the SHDS on the first four weeks of the study will be used to calculate the sample size of a larger experiment.

### Statistical analysis

The data from the pilot studies from Ghana and Vietnam will be analyzed and reported separately, though a pooled analysis will also be undertaken once both pilots have been completed. The analysis will be by intent to treat, and we do not expect to have loss of follow-up as this is a small scale pilot. Descriptive statistics and balance tables will be calculated for all variables and will be presented as medians or means with standard deviations. Differences in the outcomes between intervention and control groups will be estimated by multi-level Poisson regression models with the DDS and the SHDS as outcomes, a dummy variable for the treatment, a continuous time variable for the days and an interaction of treatment and time, and random effects at the individual level to account for the repeated measures. We will also undertake exploratory analysis for each food group of the DDS and the SHDS separately using odds ratio regressions. Socio-demographic variables that were not balanced after randomization will also be included in the robustness analysis.

A *p*-value < 0.05 will be considered statistically significant and *p*-value < 0.1 will be considered marginally significant. We will assess the need to screen outliers values greater than three standard deviations and will analyze the sensitivity of the treatment effect to the outliers.

### Data management

The data collected will be de-identified and stored on the computers of the institutions involved. Random numbers will be generated and linked to each participant, and they will be referred to exclusively by that participant code. The document linking both numbers will be destroyed once results of the study were published.

### Pilot test collaboration

The project is based on an interdisciplinary collaboration between the IFPRI, the Pennsylvania State University in partnership with the FAO, the University of Ghana, the Thai Nguyen National Hospital, the Thai Nguyen University of Medicine and Pharmacy, and the National Institute of Nutrition (NIN) of Vietnam. The principal investigator at IFPRI is responsible for coordinating the study, while the research teams based in Ghana and Vietnam recruit potential participants, send participants the informed assent and their parents the informed consent, answer questions and attend requests, and distribute the smartphones with the FRANI to eligible participants. The local research teams will also monitor participants throughout the study period, and report problems to the whole team. Because we do not anticipate negative outcomes for the participants, we have not established formal stopping rules nor an auditing conduct. The research team agreed on the final version of the pilot protocol. Weekly meetings are planned to report progress, verify deadlines, and manage the project. Any modifications in the protocol such as eligibility criteria, outcomes and analyses will be communicated to IFPRI and in-country IRBs when needed. The engineering team, based at the Pennsylvania State University, is responsible for data storage and the maintenance of FRANI. Members of all teams will be responsible for different parts of the results dissemination through journal articles, conferences and presentations.

## Discussion

This protocol describes a randomized controlled pilot study that will measure adherence, acceptability, and usability and determine the impact of an AI technology-based app in the quality of food choice of adolescent girls from Ghana and Vietnam. There is limited evidence on intervention studies that used technology to change eating habits (21). The FRANI technology has the potential to replace expensive and time-consuming questionnaires, and simplify and improve the precision of food consumption data collection. The pilot study will address gaps in the literature and advance the nutrition field by providing evidence on adherence, acceptability and usability of technology-based nutrition interventions, and by impacting the quality of food choice of adolescent girls in developing countries.

The pilot test of the FRANI is an important first step towards better quality, highly scalable data collection in nutrition and healthier food choices for a population at risk of malnutrition. The success of this intervention will depend on the use of the FRANI and the change in intake of nutritious and diverse foods. If the pilot and validation the FRANI technology are successful, the next steps in the development process include a larger randomized control trial to examine if technology-based nutrition intervention works for a more extended period of time.
